# Florfenicol Resistance in Enterobacteriaceae and Whole-Genome Sequence Analysis of Florfenicol-Resistant *Leclercia adecarboxylata* Strain R25

**DOI:** 10.1155/2019/9828504

**Published:** 2019-10-01

**Authors:** Yuanyuan Ying, Fei Wu, Chongyang Wu, Yi Jiang, Min Yin, Wangxiao Zhou, Xinyi Zhu, Cong Cheng, Licheng Zhu, Kewei Li, Junwan Lu, Teng Xu, Qiyu Bao

**Affiliations:** ^1^School of Laboratory Medicine and Life Sciences/Institute of Biomedical Informatics, Wenzhou Medical University, Wenzhou, Zhejiang 325035, China; ^2^College of Medicine and Health, Lishui University, Lishui 323000, China; ^3^Institute of Translational Medicine, Baotou Central Hospital, Baotou 014040, China

## Abstract

Due to inappropriate use, florfenicol resistance is becoming increasingly serious among animal respiratory tract and gut bacteria. To detect the florfenicol resistance mechanism among Enterobacteriaceae bacteria, 292 isolates from animal feces were examined. The agar dilution method was conducted to determine the minimum inhibitory concentration (MIC) for florfenicol, and polymerase chain reaction (PCR) was performed to detect florfenicol resistance genes. To further explore the molecular mechanism of florfenicol resistance, the whole-genome *Leclercia adecarboxylata* R25 was sequenced. Of the strains tested, 61.6% (180/292) were resistant to florfenicol, 64.4% (188/292) were positive for *floR*, and 1.0% (3/292) for *cfr*. The whole-genome sequence analysis of *L. adecarboxylata* R25 revealed that the *floR* gene is carried by a transposon and located on a plasmid (pLA-64). Seven other resistance genes are also encoded on pLA-64, all of which were found to be related to mobile genetic elements. The sequences sharing the greatest similarities to pLA-64 are the plasmids p02085-tetA of *Citrobacter freundii* and p234 and p388, both from *Enterobacter cloacae*. The resistance gene-related mobile genetic elements also share homologous sequences from different species or genera of bacteria. These findings indicate that *floR* mainly contributes to the high rate of florfenicol resistance among Enterobacteriaceae. The resistance gene-related mobile genetic elements encoded by pLA-64 may be transferred among bacteria of different species or genera, resulting in resistance dissemination.

## 1. Introduction

Enterobacteriaceae bacteria are important species that comprise the gut microbiota of domestic animals. There are reports that members of this family cause infections, for example, *Salmonella Gallinarum* as the cause of septicemia in fowl typhoid, *Escherichia coli* causing severe respiratory diseases in poultry and bovine mastitis, and septicemia in pigs due to *Klebsiella pneumoniae*. Overall, the uncontrolled use of antibiotics for the treatment and prevention of infectious diseases in animals as well as their application as growth promoters in animal husbandry has contributed to the increased spread of antimicrobial resistance genes among Enterobacteriaceae bacteria, resulting in significant economic losses [1].

Florfenicol, a synthetic broad-spectrum antibiotic derived from chloramphenicol but with better antibacterial activity and few adverse effects, has been universally used in veterinary medicine [2, 3]. However, due to inappropriate use to prevent or cure bacterial infections, florfenicol resistance has become increasingly serious and a variety of florfenicol resistance mechanisms have been characterized, including efflux pumps, rRNA methyltransferases, and chloramphenicol acetate esterases. To date, seven florfenicol resistance genes, *floR*, *cfr*, *fexA*, *fexB*, *pexA*, *optrA*, and *estDL136* [3–6], together with some variants (*floRv*, *floSt*, *cfr*(B), and *cfr*(C)) [7–9] have been discovered. Nonetheless, only a limited number of studies have reported the resistance to florfenicol or distribution of florfenicol resistance genes among Enterobacteriaceae, mainly including common species such as *Escherichia coli*, *K. pneumoniae*, *Salmonella enterica*, *Yersinia enterocolitica*, and *Proteus vulgaris* [10–14]. Moreover, the florfenicol resistance mechanisms of most Enterobacteriaceae species have not been investigated.

The genus *Leclercia* of the family Enterobacteriaceae contains only one species, *L. adecarboxylata*, which is a characteristic of a gram-negative, motile, and facultative anaerobic bacillus. First described as *Escherichia adecarboxylata* by Leclerc in 1962 [15], the species was renamed *L. adecarboxylata* by Tamura et al. in 1986 according to the recognition of its phenotypic and genotypic differences from species of the genus *Escherichia* and other species of Enterobacteriaceae [16]. *L. adecarboxylata*, which is normally present in environmental or animal sources [17], is an opportunistic human pathogen but is rarely isolated from clinical specimens. Nonetheless, it has been reported to cause bacteremia, sepsis, peritonitis, cellulitis, endocarditis, and cholecystitis in immunocompromised patients with polymicrobial infections [18]. There is no report thus far of a florfenicol molecular resistance mechanism of *L. adecarboxylata* isolated from an animal or the environment. In this work, we analyzed florfenicol resistance and the resistance genes of animal-derived Enterobacteriaceae bacteria and further examined *L. adecarboxylata* strain R25 to demonstrate the molecular resistance mechanism against florfenicol of this unique Enterobacteriaceae species.

## 2. Materials and Methods

### 2.1. Bacterial Strain and PCR Detection of Florfenicol Resistance-Associated Genes

Enterobacteriaceae strains were isolated from anal fecal samples obtained on food animal-producing farms (ducks, chickens, cows, geese, and rabbits) in Wenzhou, Zhejiang Province, China, from July 2014 to November 2015. A total of 292 Enterobacteriaceae strains were isolated. Species identification was conducted using a bioMérieux VITEK® 2 Compact Instrument (bioMérieux, Marcy L'etoile, France) and comparative analysis of 16S rRNA gene sequences from bacteria of the same genera in the National Center for Biotechnology Information (NCBI) database (https://blast.ncbi.nlm.nih.gov/Blast.cgi). Further verification of *L. adecarboxylata* R25 was conducted by homologous comparisons of the whole-genome sequences with those in NCBI. The bacterial strains and plasmids used in this study are listed in Table 1.

Bacterial genomic DNA was extracted using an AxyPrep Bacterial Genomic DNA Miniprep Kit (Axygen Scientific, Union City, CA, USA) and used as the template for subsequent PCR. PCR amplification was conducted to screen florfenicol resistance genes *floR*, *cfr*, *pexA*, *fexA*, *fexB*, and *estDL136*. Primers were designed by using Primer Premier 5.0 (Table 2). The PCR products were further confirmed by Sanger sequencing (ABI 3730 Analyzer, Foster City, CA, USA). Both strands of the PCR products were sequenced with the forward and reverse primers, and the sequencing reads were assembled with the Phred/Phrap/Consed software package (http://www.phrap.org/phredphrapconsed.html). The sequence data were compared to the NCBI nucleotide sequence database using BLAST with the max target sequences of 100, expect threshold of 10, word size of 28, max matches in a query range of 0, match scores of 1, and mismatch scores of -2 (https://blast.ncbi.nlm.nih.gov).

### 2.2. Antibiotic Susceptibility Assay

The MICs of antimicrobial agents against the 292 Enterobacteriaceae strains and corresponding recombinants carrying cloned resistance genes were determined using the standard agar dilution method recommended by the Clinical and Laboratory Standards Institute (CLSI document M100-S27, 2017). A bacterial suspension was adjusted to a turbidity equivalent to a 0.5 McFarland standard with sterilized saline solution (0.9%) and plated on Mueller-Hinton agar containing different concentrations of various antimicrobial agents. The plates were incubated at 37°C for 24 h. The MIC was recognized as the lowest antibiotic concentration resulting in no colony growth. Each of the tests was carried out in triplicate. *E. coli* ATCC 25922 was used as a quality control strain. The resistance breakpoints of Enterobacteriaceae for florfenicol were set referring to those for chloramphenicol in the guidelines of CLSI document M100-S27 (2017).

### 2.3. Whole-Genome Sequencing

A 20 kb library was generated using a SMRTbell Template Prep Kit (Pacific Biosciences, Menlo Park, CA, United States) according to the PacBio standard protocol and sequenced using a PacBio RS II instrument. In addition, an Illumina library with 300 bp insert sizes was constructed and sequenced from both ends using the HiSeq 2500 platform (both PacBio RS II and HiSeq 2500 sequencing were carried out at Annoroad Gene Technology Co. Ltd., Beijing, China). Reads of the clean data derived from the raw data of HiSeq 2500 sequencing were initially assembled de novo with the SOAPdenovo software to obtain contigs of the genome sequences. PacBio long reads were assembled using Canu software [19]. Two FASTQ sequence files corresponding to the reads derived from HiSeq 2500 sequencing were used to control assembly quality and to correct possibly misidentified bases. Potential ORFs were predicted using Glimmer software (http://ccb.jhu.edu/software.shtml) and annotated against a nonredundant protein database using BLASTX (https://blast.ncbi.nlm.nih.gov). Plasmid typing was performed using BLAST in the PlasmidFinder database (https://cge.cbs.dtu.dk/services/MLST/).

### 2.4. Cloning of Resistance Genes

The primers used to clone candidate genes with potential upstream promoter regions are shown in Table 2. PrimeSTAR HS DNA Polymerase (TaKaRa, Dalian, China) was used to amplify resistance genes according to the manufacturer's instructions. A poly(A) tail was added to each purified PCR product (prom-ORF) using the DNA A-Tailing Kit (TaKaRa, Dalian, China), and the fragment was then cloned into the pMD™19-T vector (TaKaRa, Dalian, China). The resulting recombinant plasmid (pMD™19-T-prom-ORF) was transformed into *E. coli* DH5*α* using the calcium chloride method. Transformants were selected on LB agar plates containing 100 *μ*g/mL ampicillin. The cloned PCR product was further confirmed by Sanger sequencing.

### 2.5. Comparative Genomic Analysis

The plasmid and chromosome genome sequences used in this study were downloaded from NCBI (http://www.ncbi.nlm.nih.gov). Comparisons of nucleotide and amino acid sequences were performed using BLASTN and BLASTP, respectively. The map of the plasmid with GC content and GC skew was drawn using the online CGView Server (http://stothard.afns.ualberta.ca/cgview_server/) and local GView 1.7 with visual interface [20]. Family classification and domain prediction of mobile genetic elements (MGEs) were verified by comparison using the InterPro database (http://www.ebi.ac.uk/interpro/) and ISfinder database (https://isfinder.biotoul.fr/). Other bioinformatics tools were utilized through Perl and BioPerl (http://www.perl.org/).

## 3. Results and Discussion

### 3.1. Animal Enterobacteriaceae Bacteria and Their Resistance to Phenicols

Florfenicol as well as tetracyclines, beta-lactams, and trimethoprim/sulfonamides is widely used to treat animal infections. The resistance rates for these antibiotics have increased greatly, and the emergence of multidrug-resistant bacteria is increasing [2]. In this work, we detected resistance to florfenicol and chloramphenicol among 292 Enterobacteriaceae strains isolated from fecal specimens of 5 types of animals (rabbit, chicken, cow, goose, and duck). The strains isolated belong to 11 genera: *Escherichia* (86.0%, 251/292), *Shigella* (0.7%, 2/292), *Klebsiella* (2.7%, 8/292), *Serratia* (0.3%, 1/292), *Proteus* (3.1%, 9/292), *Citrobacter* (1.7%, 5/292), *Enterobacter* (3.4%, 10/292), *Yersinia* (0.3%, 1/292), *Leclercia* (0.3%, 1/292), *Pantoea* (1.0%, 3/292), and *Kluyvera* (0.3%, 1/292) ([Supplementary-material supplementary-material-1]). The overall resistance rates to florfenicol and chloramphenicol were 61.6% and 65.1%, respectively. Except for the 10 strains of *Enterobacter*, which exhibited low resistance rates of 20.0% to both florfenicol and chloramphenicol, all the other strains from various genera showed high resistance rates of 50.0-88.9% to florfenicol and 64.1-100.0% to chloramphenicol. *Proteus* spp. exhibited the highest resistance rate of 88.9% (8/9) to florfenicol; *Klebsiella* spp. and *Escherichia* spp. also displayed high resistance rates of 75.0% (6/8) and 62.5% (157/251) to florfenicol, respectively (Table 3).

The rates of resistance to florfenicol among the Enterobacteriaceae bacteria isolated in this work appeared to be much higher than those reported for bacteria from the same or different genera or families. For example, in one study, isolates of *Pasteurella multocida*, *Actinobacillus pleuropneumoniae*, and *Streptococcus suis* from cattle and pig respiratory tract infections showed resistance rates to florfenicol of <1% [21]. Another report demonstrated that the rates of florfenicol resistance of *A. pleuropneumoniae* and *P. multocida* isolated from pig respiratory tract infections were 2.0% and 6.0%, respectively [22]. Resistance rates of 25.4% and 15.3% to florfenicol have also been reported for *Salmonella* and *Yersinia*, respectively [13, 23]. In addition, *E. coli* strains from canine urinary tract infections showed higher resistance, at a rate of 31.6% (36/114), to florfenicol than did other pathogens [10]. More recently, a resistance level to chloramphenicol for *Staphylococcus pseudintermedius* of 32 *μ*g/mL (MIC90) was reported, but no resistance to florfenicol was detected in this species (MIC90 = 4 *μ*g/mL) [24]. Despite these publications, there is no report thus far regarding the resistance to florfenicol for other species of Enterobacteriaceae. In this work, except for four genera with only 1 (*Yersinia*, *Serratia*, and *Kluyvera*) or 3 (*Pantoea*) isolates that were sensitive to florfenicol, the other 7 genera tested exhibited some degree of resistance to florfenicol. In particular, this is the first report of resistance to florfenicol among isolates of *Shigella* (1/2), *Klebsiella* (6/8), *Proteus* (8/9), *Citrobacter* (5/5), *Enterobacter* (2/10), and *Leclercia* (1/1) isolated from animals.

### 3.2. Distribution of Florfenicol Resistance Genes among Animal Enterobacteriaceae Isolates

Seven florfenicol resistance genes (*floR*, *fexA*, *fexB*, *pexA*, *cfr*, *optrA*, and *estDL136*) have been identified among bacteria. Our PCR screening of florfenicol resistance genes among 292 Enterobacteriaceae isolates revealed positive results for only *floR* and *cfr*. Among the strains, 64.4% (188/292) were positive for *floR*, whereas *cfr* was only identified in three *Proteus* strains (1.0%, 3/292) isolated from geese. No isolates were positive for *pexA*, *fexA*, *fexB*, *optrA*, or *estDL136* (Table 3). With the exception of *Enterobacter* isolates exhibiting a low rate of positivity for the *floR* gene (30.0%), the other genera showed high *floR*-positive rates of 50.0-88.9%, in accordance with the florfenicol resistance rates within each genus. *Proteus* spp. exhibited the highest rates of resistance gene positivity, at 88.9% (8/9) for *floR* and 33.3% (3/9) for both *floR* and *cfr*. Of the seven florfenicol resistance genes, *floR* is the main and most common florfenicol resistance gene identified in both gram-positive and gram-negative bacteria [25] and the only one identified in *K. pneumoniae* strains originating from both humans and animals [11, 26]. In our study, the rate of *floR* positivity for *K. pneumonia* was 75.0% (6/8), much higher than the 7.0% (23/328) of human clinical *K. pneumonia* isolated from the same district [11] and indicating the wide use of florfenicol in local animal farming.

The rates of *floR* gene positivity from various bacteria differed significantly. A high rate of *floR* gene positivity (81.3%) has been reported for clinical *Vibrio cholerae* isolates from some Iranian provinces [27]. Regarding *Salmonella* isolates from broiler farms in East China, the overall rate of *floR* gene positivity was 43.5%, and it is interesting that the rates between serotypes differed greatly. *Salmonella enterica* serovar Indiana isolates displayed a positive rate up to 96.2% (128/133), though that of *S. enterica* serovar Enteritidis strains was only 3.9% (7/177) [12]. Among gram-negative bacteria, the *cfr* genes have been found in *P. vulgaris* (as in this work) and in *E. coli* [6, 14]. Moreover, *floRv* and *floSt*, variants of *floR*, have only been identified in a few gram-negative bacteria, including *Stenotrophomonas maltophilia* [8] and *Salmonella* [7], respectively. In contrast, *estDL136* has only been identified in *E. coli* [6]. *fexA*, *fexB*, *pexA*, *optrA*, and *cfr* were mainly harbored by gram-positive bacteria [4, 5, 28], though *fexA* and *pexA* have been found in *E. coli* [6].

### 3.3. Sequencing Analysis of the *L. adecarboxylata* R25 Genome

The *floR* gene has been found on the chromosome as well as plasmids. It was first identified on the chromosome of *Salmonella typhimurium* DT104 [29] and later on a plasmid of *E. coli* isolate BN10660 [30] and IncC plasmid R55 of *K. pneumoniae* [26], among others. However, no publication has reported the resistance level or the resistance mechanism of *L. adecarboxylata* to florfenicol. Thus, to elucidate the florfenicol resistance mechanism of *L. adecarboxylata*, we sequenced an *L. adecarboxylata* isolate designated as R25 isolated from rabbit feces with high MICs to florfenicol (128 *μ*g/mL) and chloramphenicol (128 *μ*g/mL) (Table 4). Although three complete genomes of the same species of *L. adecarboxylata* are available in the NCBI nucleotide database, no *floR* gene was identified among them. Of these three *L. adecarboxylata* isolates, only one, USDA-ARS-USMARC-60222 (CP013990.1), without a plasmid was from an animal (calf). The other two, LSNIH3 (CP026387.1) and LSNIH1 (CP026167.1), were from the hospital environment of housekeeping closet drains in the United States.

**Figure 1:**
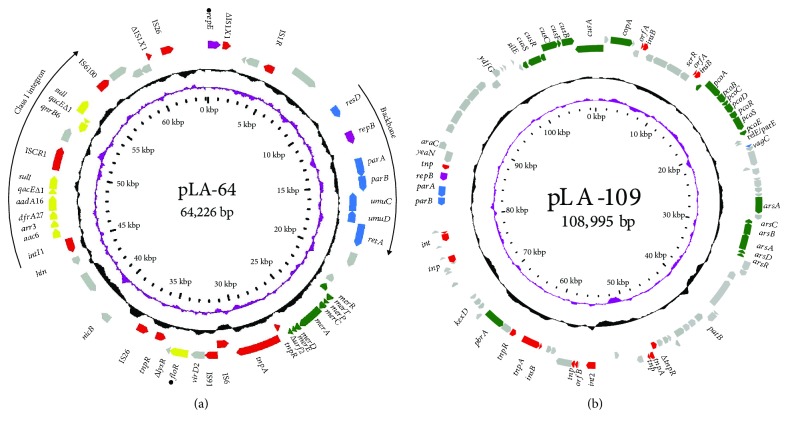
Genomic structure of the plasmids pLA-64 (a) and pLA-109 (b). Genes are denoted by arrows and are colored based on gene function classification. Counting from the outside toward the center: (1) genes encoded on the leading strand (outwards) or the lagging strand (inwards) with the hypothetical protein left blank; (2) an average G+C content of 50%, whereas a G+C content of more than 50% is shown toward the outside and a G+C content of less than 50% toward the inside; (3) GC skew (G–C/G+C) with a positive GC skew toward the outside and a negative GC skew toward the inside; and (4) scale in bp. Genes with different functions are shown in different colors: red: transposable elements; yellow: drug resistance; green: heavy metal resistance; blue: backbone; purple: replication; gray: genes with other functions.

The genome of *L. adecarboxylata* R25 consists of a 4.74 Mb circular chromosome encoding 4,293 open reading frames (ORFs) and two plasmids, pLA-64 (64,226 bp) and pLA-109 (108,995 bp) encoding 82 and 121 ORFs, respectively (Figures 1(a) and 1(b) and Table 5). Comparative genomic analysis showed that the genomes of the three *L. adecarboxylata* strains (USDA-ARS-USMARC-60222, CP013990.1; LSNIH3, CP026387.1; and LSNIH1, CP026167.1) share the highest chromosome sequence identities with that of *L. adecarboxylata* R25, at 82%, 81%, and 81% coverage and 94%, 93%, and 92% identity, respectively.

Annotation of the complete genome of *L. adecarboxylata* R25 revealed that it encodes nine drug resistance genes, conferring resistance to chloramphenicols (*floR* and *mdfA*), aminoglycosides (*aac(6*′)-*Ib-cr* and *aadA16*), quinolones (*qnrB6*), sulfonamides (*sul1*), trimethoprim (*dfrA27*), rifampicin (*arr-3*), and a quaternary ammonium compound (*qacEΔ1*). Five resistance genes (*floR*, *aac(6*′*)-Ib-cr*, *arr-3*, *aadA16*, and *qnrB6*) with their promoter regions in *L. adecarboxylata* R25 were cloned, and expression of these resistance genes conferred resistance to the corresponding antibiotics in comparison with the MIC levels of the control strain DH5*α*. Expression of the *floR* gene resulted in a 3-fold increase in resistance to florfenicol, with the *aac(6*′*)-Ib-cr* and *aadA16* genes conferring at least 3- to 6-fold increase in resistance to amikacin, kanamycin, streptomycin, and spectinomycin. For the *qnrB6* gene expression, at least a 2-fold increase in resistance to nalidixic acid, norfloxacin, and ciprofloxacin was achieved and a 4-fold increase in resistance to rifampin was obtained with *arr-3* (Table 4). The resistance gene profile and MIC results for the cloned resistance genes against florfenicol, chloramphenicol, rifampin, and spectinomycin were in accordance with the host's resistance phenotypes (Table 4). A few publications have demonstrated that in nonclinical *L. adecarboxylata* isolates, several resistance genes (such as *rmtB*, *qnrA*, *Oqxb*, and *qep*) mediate resistance to certain antibiotics, including aminoglycosides and fluoroquinolone [17, 31]. Resistance to beta-lactams among clinical *L. adecarboxylata* isolates can be ascribed to the acquisition of resistance plasmids carrying *bla*_SHV-12_ and *bla*_NDM-1_ [32], but these isolates are generally sensitive to commonly used antibiotics such as tetracyclines, aminoglycosides, quinolones, and chloramphenicol [33]. The *floR*, *aadA16*, and *qnrB6* genes identified in *L. adecarboxylata* R25 have not been reported previously.

### 3.4. Comparative Analysis of the Resistance Plasmid pLA-64 and Resistance Gene-Related Sequences

Among 9 resistance genes, only 1 (*mdfA*) is located on the chromosome, whereas the other 8 (*floR*, *aac(6*′*)-Ib-cr*, *arr-3*, *dfrA27*, *aadA16*, *qacEΔ1*, *sul1*, and *qnrB6*) are encoded by the plasmid pLA-64 (Figure 1(a)). In addition to drug resistance genes, the complete sequence encodes four clusters of heavy metal resistance genes, with one mercury resistance gene cluster on pLA-64 (Figure 1(a)) and the other three (copper, copper/silver, and arsenate resistance gene clusters) on plasmid pLA-109 (Figure 1(b)).

**Figure 2:**
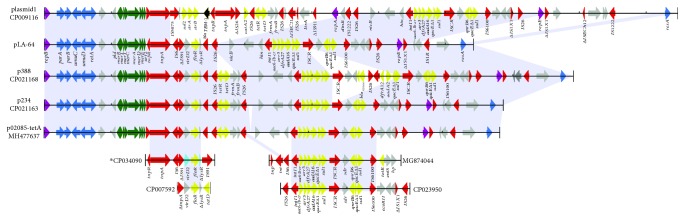
Comparative genomic analysis of pLA-64 and homologous sequences from other plasmids. plasmid1 (CP009116.1), from a *K. pneumoniae* strain isolated from the human; p388 (CP021168.1), from *E. cloacae* strain 388 isolated from the human; p234 (CP021163.1), from *E. cloacae* strain 234 isolated from the human; p02085-tetA (MH477637.1), from *C. freundii* strain 1509-02085 without origin information; CP034090.1, the chromosome of *P. mirabilis* strain PmSC1111 isolated from swine; CP007592.1, the chromosome of *E. coli* O157:H16 Santai isolated from the duck; MG874044.1, plasmid pSa76-CIP of *Salmonella* sp. without origin information; and CP023950.1, plasmid unnamed3 of *K. pneumoniae* FDAARGOS_447 isolated from the human. Genes with different functions are shown in different colors: red: transposable elements; yellow: drug resistance; green: heavy metal resistance; blue: backbone; purple: replication; gray: genes with other functions.

The sequences sharing the greatest similarities to pLA-64 are the three plasmids p02085-tetA (MH477637.1) of *C. freundii* strain 1509-02085 (no original information) and p234 (CP021163.1) and p388 (CP021168.1) from *E. cloacae* strains isolated from humans. These plasmids encompass nearly the entire sequence of pLA-64, with more than 99% coverage and 99% identity. p02085-tetA is 68 kb in length and p234 69 kb, only 4 and 5 kb longer than pLA-64, respectively, with an extra resistance gene-related fragment (encoding *tetR*-*tetD*-*frmA*-*frmB*-IS*26*) inserted at position 37 kb of pLA-64. p388 is 79 kb in size and 15 kb longer than pLA-64, with two extra resistance gene-related fragments inserted at positions 37 kb and 51 kb of pLA-64, respectively. The plasmid with low similarity to pLA-64 is plasmid1 (CP009116.1) from a human clinical *K. pneumoniae* strain; it is 95 kb in length (31 kb longer than pLA-64) and contains 81% (52/64) of the sequence of pLA-64 but without the *floR* gene-related region (encoding IS*6*-*Δ*IS*91*-*virD2*-*floR*-*ΔlysR*) (Figure 2). All these plasmids belong to the same Inc group carrying two replicons: FIA(HI1) and R. However, no plasmids from the two *Leclercia* strains LSNIH3 (CP026387.1) and LSNIH1 (CP026167.1) share sequence identity of more than 28% with pLA-64. The plasmids in these two strains harbor the replicons of other Inc groups, including N, FII (pCTU2), HI1A (CIT), and HI1B (CIT), as opposed to the FIA (HI1) and R of pLA-64. These findings indicate that pLA-64 homologous plasmids may transfer among bacteria of different genera of various (animal and human) origins.

The plasmid pLA-64 consists roughly of two parts: a backbone and a variable region. The backbone is composed of segments responsible for replication (*repBE*), DNA repair (*umuCD*), and plasmid maintenance (*parAB*), whereas the variable regions harbor a number of MGEs, such as insertion sequences, transposons, and an integron. All eight resistance genes encoded by the pLA-64 are related to MGEs. Six are found in a class 1 integron (*intl1*-*aac(6*′)-*Ib*-*cr*-*arr-3-dfrA27-aadA16-qacEΔ1*-*sul1*); the other two (*qnrB6* and *floR*) are related to transposons. The *floR* gene is located in a fragment approximately 7.6 kb in length encoding the Tn*3*-IS*6*-*Δ*IS*91*-*virD2*-*floR*-*ΔlysR* gene cluster (Figures 1(a) and 2). In addition to the plasmids (p02085-tetA, p234, p388, and plasmid1) mentioned above, sequences with higher identities with resistance gene-related MGEs were identified in other plasmid or chromosome sequences, such as those encoded by the chromosome of *Proteus mirabilis* strain PmSC1111 (CP034090.1), the chromosome of *E. coli* O157:H16 Santai (CP007592.1), *Salmonella* sp. plasmid pSa76-CIP (MG874044.1), and plasmid unnamed3 of *K. pneumoniae* FDAARGOS_447 (CP023950.1) (Figure 2). The gene array of the class 1 integron has also been identified in other animal bacteria of different species, such as *E. coli* and *Enterobacter amnigenus* isolated from swine feces and swine farm wastewater, respectively [34]. Similarly, the *qnrB6*-related gene cluster was identified on *K. pneumoniae* plasmid pLC24 isolated from dog vomit [35]. Although the genetic environment of the *floR* gene encoded on the chromosome or plasmid from different bacteria differed, the gene cluster of *virD2*-*floR*-*ΔlysR* encoded on pLA-64 in this work is conserved in most cases [30].

## 4. Conclusion

The results of this work show high rates of resistance to florfenicol (61.6%, 180/292) and chloramphenicol (65.1%, 190/292) among animal Enterobacteriaceae isolates. The *floR* gene is common among various species (64.4%. 188/292), though *cfr* was only identified in some *Proteus* spp. (1.0%, 3/292). All resistance genes including *floR* encoded on the plasmid pLA-64 in *L. adecarboxylata* R25 are related to MGEs. Comparative genomic analysis demonstrated that the sequences sharing the greatest similarities to pLA-64 are three plasmids from *C. freundii* and *E. cloacae* strains isolated from humans. These findings indicate a high rate of florfenicol resistance among local animal bacteria, with the *floR* gene also being highly prevalent. Resistance plasmids may be transferred between bacteria of different species or genera and of different (animal and human) origins and cause resistance dissemination.

### 4.1. Accession Numbers

The complete nucleotide sequences of the chromosome and plasmids have been submitted to the NCBI database, and the accession numbers of chromosome, pLA-64, and pLA-109 are CP035382.1, CP035381.1, and CP035380.1, respectively.

## Figures and Tables

**Table 1:** Bacterial strains and plasmids used in this study.

Strains and plasmids	Description	Source
Strain		
*L. adecarboxylata* R25	Multiresistant isolate derived from rabbits	This study
*E. coli* DH5*α*	Used as a host for cloning of PCR products	Our lab collection
*E. coli* ATCC25922	Used as a control strain	Our lab collection
pMD™19-T-ORFs*/E. coli* DH5*α*	*E. coli* DH5*α* carrying the recombinant plasmid pMD™19-T carrying resistance gene ORFs and promoter regions (*aac(6*′)-*Ib*-*cr*, *aadA16*, *arr-3*, *qnrB6*, and *floR*)	This study
Plasmid		
pMD™19-T	Cloning vector for the PCR products of all resistance genes and its promoter region, Amp^r^	This study

Abbreviations: Amp: ampicillin; r: resistance.

**Table 2:** Primers used in this work.

Primer	Sequence (5′-3′)	Purpose	Product length (bp)	Annealing temperature (°C)
27F	AGAGTTTGATCCTGGCTCAG	16S rRNA	1465	55
1492R	TACGGCTACCTTGTTACGACTT			
*floR*-F	ATGGTGATGCTCGGCGTGGGCCA	*floR* gene screening	800	58
*floR*-R	GCGCCGTTGGCGGTAACAGACACCGTGA			
*cfr*-F	GGGAGGATTTAATAAATAATTTTGGAGAAACAG	*cfr* gene screening	580	58
*cfr*-R	CTTATATGTTCATCGAGTATATTCATTACCTCATC			
*pexA*-F	CTTCCAGTTGAGAAGCGAGC	*pexA* gene screening	319	56
*pexA*-R	AGAAGCATACCCGTGAACATG			
*fexA*-F	CTCTTCTGGACAGGCTGGAA	*fexA* gene screening	332	57
*fexA*-R	CCAGTTCCTGCTCCAAGGTA			
*fexB*-F	ACTGGACAGGCAGGCTTAAT	*fexB* gene screening	319	57
*fexB*-R	CCTGCCCCAAGATACATTGC			
*optrA*-F	CTTATGGATGGTGTGGCAGC	*optrA* gene screening	309	56
*optrA*-R	CCATGTGGTTTGTCGGTTCA			
*estDL136*-F	ATGCCGTTAAACCCCCATGTCGAAG	*estDL136* gene screening	933	55
*estDL136*-R	TCAAGCGAGGTCTCTTTTAAGATT			
pro-*aac(6*′)-*Ib*-*cr*-F	GCTATCAGGTCAAGTCTGCTTTTATT	*aac(6*′)-*Ib*-*cr* gene cloning	674	62
pro-*aac(6*′)-*Ib*-*cr*-R	TTAGGCATCACTGCGTGTTCGCTCG			
pro-*aadA16*-F	GTGTTTCCATCTATAGAAGCAGCAATG	*aadA16* gene cloning	1274	56
pro-*aadA16*-R	TTAAGCTGCGCCGCGAAGCGGCGTC			
pro-*arr-3*-F	GGTGACCAACAGCAACGATTCCGTCAC	*arr-3* gene cloning	1104	62
pro-*arr-3*-R	CTAGTCTTCAATGACGTGTAAACCAC			
pro-*qnrB6*-F	GTTATTATGCACGGCTTACAGCAGGCAA	*qnrB6* gene cloning	849	62
pro-*qnrB6*-R	CTAACCAATCACCGCGATGCCAAGCCG			
pro-*floR*-F	GTTGCGAAGCAAAAGATAATCGGATAAA	*floR* gene cloning	1415	62
pro-*floR*-R	TTAGACGACTGGCGACTTCTCGGTGGCA			

**Table 3:** Prevalence and antibiotic resistance of Enterobacteriaceae isolated from animal feces from animal farms in South China.

Genera	Isolates	Resistance^a^						Resistance genes						
		Florfenicol^b^			Chloramphenicol			*floR*	*cfr*	*pexA*	*fexA*	*fexB*	*optrA*	*estDL136*
		S	I	R	S	I	R							
*Escherichia*	251 (86.0%)	87 (34.7%)	7 (2.8%)	157 (62.5%)	84 (33.5%)	6 (2.4%)	161 (64.1%)	164 (65.3%)	0	0	0	0	0	0
*Klebsiella*	8 (2.7%)	2 (25.0%)	0	6 (75.0%)	0	0	8 (100.0%)	6 (75.0%)	0	0	0	0	0	0
*Proteus*	9 (3.1%)	1 (11.1%)	0	8 (88.9%)	0	0	9 (100.0%)	8 (88.9%)	3 (1.0%)	0	0	0	0	0
*Enterobacter*	10 (3.4%)	7 (70.0%)	1 (10.0%)	2 (20.0%)	8 (80.0%)	0	2 (20.0%)	3 (30.0%)	0	0	0	0	0	0
Other genera^c^	14 (4.8%)	7 (50.0%)	0	7 (50.0%)	4 (28.6%)	0	10 (71.4%)	7 (50.0%)	0	0	0	0	0	0
Total	292 (100.0%)	104 (35.6%)	8 (2.7%)	180 (61.6%)	96 (32.9%)	6 (2.1%)	190 (65.1%)	188 (64.4%)	3 (1.0%)	0	0	0	0	0

Abbreviations: S: sensitive; I: intermediate; R: resistance. ^a^Criteria as published by CLSI 2017. ^b^Using chloramphenicol breakpoints. ^c^Other genera of Enterobacteriaceae bacteria including 2 *Shigella*, 1 *Serratia*, 5 *Citrobacter*, 1 *Yersinia*, 1 *Leclercia*, 3 *Pantoea*, and 1 *Kluyvera*.

**Table 4:** MICs of antibiotics for the *L. adecarboxylata* R25 strain and its derivatives (*μ*g/mL).

Strain	FFC	CHL	RIF	AMK	GEN	STR	SPE	KAN	NEO	NAL	NOR	CIP
*L. adecarboxylata* R25	128	128	256	2	0.125	8	64	4	0.5	8	1	0.25
pMD™19-T-*aac(6*′)-*Ib*-*cr/E. coli* DH5*α*	8	8	16	16	0.25	4	8	64	2	4	0.13	<0.03
pMD™19-T-*aadA16*/*E. coli* DH5*α*	8	8	16	2	0.25	>64	>64	1	2	4	<0.03	<0.03
pMD™19-T-*arr-3*/*E. coli* DH5*α*	8	8	512	2	0.25	4	8	1	2	4	<0.03	<0.03
pMD™19-T-*qnrB6*/*E. coli* DH5*α*	8	8	16	2	0.25	4	8	1	2	32	0.25	0.25
pMD™19-T-*floR*/*E. coli* DH5*α*	64	32	16	2	0.25	4	8	2	2	4	<0.03	<0.03
*E. coli* DH5*α*	8	8	32	2	0.25	4	8	1	2	4	0.06	<0.03
*E. coli* ATCC25922	4	4	8	4	0.5	8	8	4	2	2	<0.03	<0.03

Abbreviations: FFC: florfenicol; CHL: chloramphenicol; RIF: rifampin; AMK: amikacin; GEN: gentamicin; STR: streptomycin; SPE: spectinomycin; KAN: kanamycin; NEO: neomycin; NAL: nalidixic acid; NOR: norfloxacin; CIP: ciprofloxacin.

**Table 5:** General features of the *L. adecarboxylata* R25 genome.

	Chromosome	pLA-64	pLA-109
Size (bp)	4,741,546	64,226	108,995
GC content (%)	56.43	53.04	50.14
Open reading frames (ORFs)	4,293	82	121
Known proteins	3355 (79.2%)	50 (62.5%)	79 (70.5%)
Hypothetical proteins	882 (20.8%)	30 (37.5%)	33 (29.5%)
Protein coding	87.31%	80.43%	77.95%
Average ORF length (bp)	958	645	758
Average protein length (aa)	977	214	251
tRNAs	85	0	0
rRNA operons	7∗(16s-23s-5s) 1∗(16s-23s-5s-5s)	0	0

## Data Availability

The data used to support the findings of this study are available from the corresponding author upon request.

## Supplementary Materials

Supplementary Materials Table S1: distribution of Enterobacteriaceae isolated from the animal feces specimens from the animal farms in South China.
