# Supplementary material for: Florfenicol Resistance in Enterobacteriaceae and Whole-Genome Sequence Analysis of Florfenicol-Resistant Leclercia adecarboxylata Strain R25
Source: Int J Genomics. 2019 Oct 1;2019:9828504. doi: 10.1155/2019/9828504 (PMC6791223; doi:10.1155/2019/9828504)
Supplement: Supplementary Materials — Table S1: distribution of Enterobacteriaceae isolated from the animal feces specimens from the animal farms in South China. [file 9828504.f1.doc]

TableS1 Distribution of Enterobacteriaceae isolated from the animal feces specimens from the animal farms in South China

| Genera | Rabbit | Chicken | Cow | Goose | Duck | Total |  |
| --- | --- | --- | --- | --- | --- | --- | --- |
| *Escherichia* | 37 (75.5%) | 106 (91.4%) | 64 (92.8%) | 26 (78.8%) | 18 (72.0%) | 251 (86.0%) |  |
| *Shigella* | 1 (2.0%) | 0 | 0 | 1 (3.0%) | 0 | 2 (0.7%) |  |
| *Klebsiella* | 4 (8.2%) | 0 | 3 (4.3%) | 0 | 1 (4.0%) | 8 (2.7%) |  |
| *Serratia* | 1 (2.0%) | 0 | 0 | 0 | 0 | 1 (0.3%) |  |
| *Proteus* | 0 | 3 (2.6%) | 0 | 6 (18.2%) | 0 | 9 (3.1%) |  |
| *Citrobacter* | 4 (8.2%) | 1 (0.9%) | 0 | 0 | 0 | 5 (1.7%) |  |
| *Enterobacter* | 0 | 3 (2.6%) | 2 (2.9%) | 0 | 5 (20.0%) | 10 (3.4%) |  |
| *Yersinia*. | 1 (2.0%) | 0 | 0 | 0 | 0 | 1 (0.3%) |  |
| *Leclercia* | 1 (2.0%) | 0 | 0 | 0 | 0 | 1 (0.3%) |  |
| *Pantoea* | 0 | 3 (2.6%) | 0 | 0 | 0 | 3 (1.0%) |  |
| *Kluyvera* | 0 | 0 | 0 | 0 | 1 (4.0%) | 1 (0.3%) |  |
| Total | 49 (16.8%) | 116 (39.7%) | 69 (23.6%) | 33 (11.3%) | 25 (8.6%) | 292 (100.0%) |  |
